# Identification of risk-factors for the development of depressive symptoms in perinatal period: A longitudinal study

**DOI:** 10.1192/j.eurpsy.2021.312

**Published:** 2021-08-13

**Authors:** A. Vece, G. Tarantino, M. Carfagno, M. Raia, C. Ciampi, M. Di Vincenzo, A. Pitocco, G. Sampogna, M. Luciano, M. Torella, A. Fiorillo

**Affiliations:** 1 Psychiatry, University of Campania “Luigi Vanvitelli”, napoli, Italy; 2 Ginecology And Obstetrics, University of Campania “Luigi Vanvitelli”, napoli, Italy

**Keywords:** Perinatal depression, depressive symptoms, risk-factors, longitudinal study

## Abstract

**Introduction:**

Perinatal depression is a severe and disabling condition, which affects negatively both mothers’ and children’s mental health and well-being. About 12.8% of pregnant women report depressive symptoms in the perinatal period.

**Objectives:**

The aims of the present study are to: 1) identify factors (socio-demographic and clinical) associated with an increased risk of developing PD; 2) promote a screening program on PD.

**Methods:**

All pregnant women were assessed at each trimester of pregnancy, three days after the childbirth and after 1, 3, 6 and 12 months, with the Edinburgh Postpartum Depression Scale (EPDS). Women scoring ≥10 on the EPDS were invited to receive a full psychiatric evaluation to confirm the diagnosis.

**Results:**

420 women were recruited. 52.9%, 27.6% and 31.6% of participants presented an EPDS≥ 10 score at The I, II and III trimester of pregnancy, respectively. The percentage of patients with and EPS score ≥19 is 16.6%, 6.8%, 6.8%, 11.3% and 7.8% in 3 days following the childbirth and after 3, 6, 9 and 12 months, respectively. Higher EPDS scores are predicted by the presence of anxiety symptoms before pregnancy and of depressive and anxiety symptoms in previous pregnancies (p<0.05). Women with family conflicts and with anxiety symptoms in the partner are more likely to report higher EPDS scores (p<0.001).

**Conclusions:**

Our results confirm that perinatal depression is a highly prevalent condition. An early identification of depressive symptoms during this period is crucial in order to reduce the long-term negative impact on the mothers, the newborn and other family members.

**Disclosure:**

No significant relationships.

